# Supplementary material for: Effectiveness of Preoperative Chest Physiotherapy in Patients Undergoing Elective Cardiac Surgery, a Systematic Review and Meta-Analysis
Source: Medicina (Kaunas). 2022 Jul 8;58(7):911. doi: 10.3390/medicina58070911 (PMC9319848; doi:10.3390/medicina58070911)
Supplement: Supplementary file 1 [file medicina-58-00911-s001.zip › medicina-1783175-supplementary.pdf]

Supplementary Materials

Cochrane database: in the search box, we typed the keywords, and the search was run. After choosing to apply the search in the text of the articles, we excluded Cochrane reviews, protocols, editorials, special collections, and clinical answers. We selected articles in English only, and filtered out records of articles published before 2000. Finally, 11889 records were obtained.

PubMed Central: the keywords were types into the search box, the search was specified to involve the whole text of the articles, and the filters were applied to restrict the search beginning from 1 January 2000. Finally, this search resulted in 6605 available articles.

Embase: in the Embase library, we applied a quick search, limiting the date to beginning date to 1 January 2000, the source of search to Embase, the language to English, and the article type to original articles. Finally, 5568 articles were obtained.
